# Supplementary material for: Chromatin reorganization drives overexpression of a Btaf1 variant underpinning hematopoietic aging
Source: Nat Commun. 2026 Mar 18;17:4129. doi: 10.1038/s41467-026-70787-4 (PMC13149550; doi:10.1038/s41467-026-70787-4)
Supplement: Supplementary file 1 — Supplementary Information [file 41467_2026_70787_MOESM1_ESM.pdf]

**Chromatin reorganization drives overexpression of a *Btaf1* variant underpinning hematopoietic aging**

Le Zong[1,†], Bongsoo Park[1,†], Yaqiang Cao[2], Fei Ma[3], Ferda Tekin-Turhan[1], Wakako Kuribayashi[1], Keji Zhao[2] & Isabel Beerman[1,*]

[1]Epigenetics and Stem Cell Unit, Translational Gerontology Branch, National Institute on Aging, NIH, Baltimore, MD, USA

[2]Laboratory of Epigenome Biology, Systems Biology Center, National Heart, Lung, and Blood Institute, NIH, Bethesda, MD, USA

[3]Laboratory of Molecular Biology and Immunology, National Institute on Aging, NIH, Baltimore, MD, USA

[†]Equally Contributing Authors

*Correspondence: isabel.beerman@nih.gov

# Supplementary Fig. 1

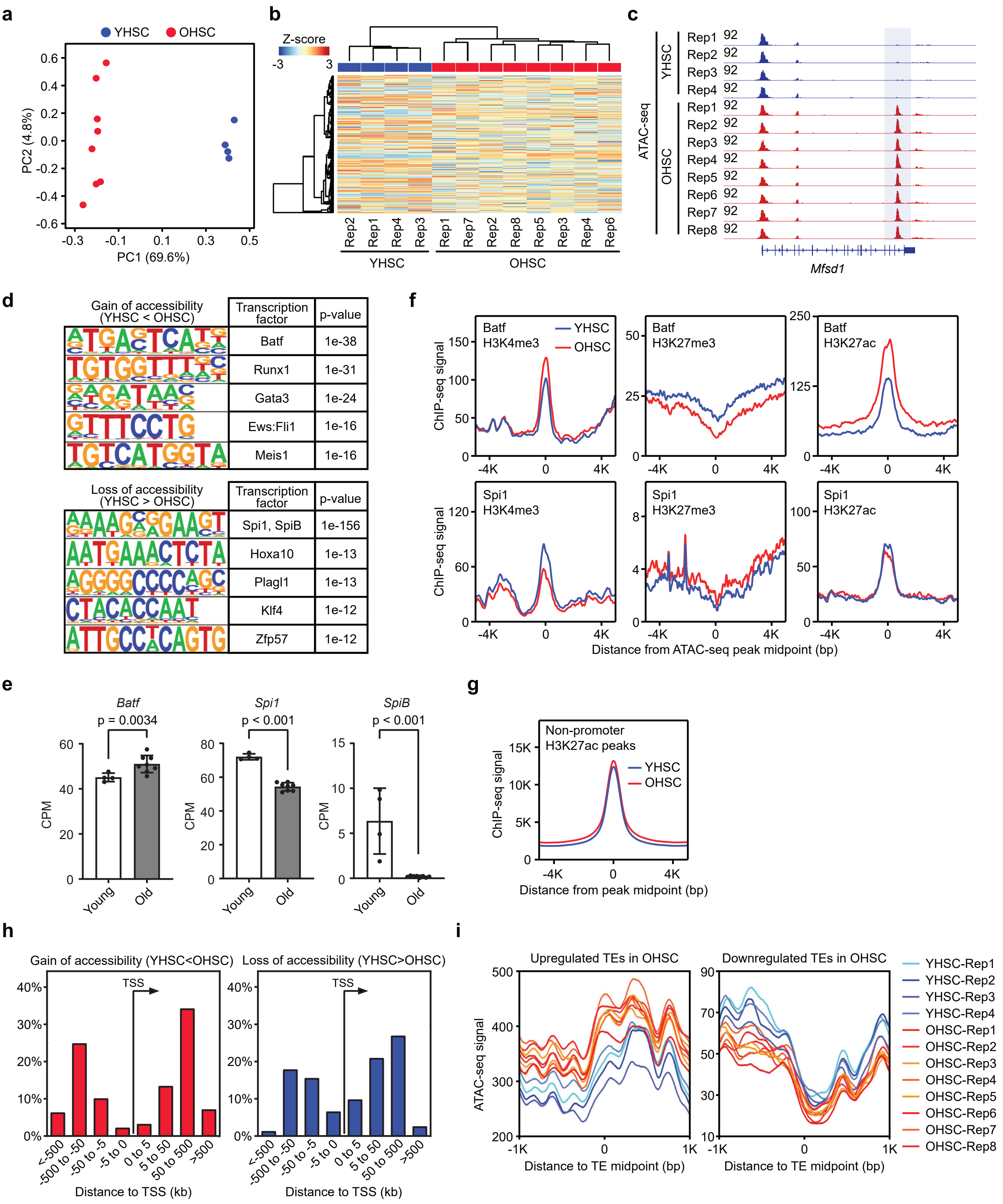

**Supplementary Fig. 1: Analysis of chromatin accessibility and enhancer activity in young and old HSCs.**

(**a**) PCA plot of ATAC-seq datasets from 4 young and 8 old HSC samples.

(**b**) Unsupervised hierarchical clustering of ATAC-seq datasets from 4 young and 8 old HSC samples.

(**c**) IGV tracks of an example DAR at *Mfsd1* locus. YHSC (n = 4), OHSC (n = 8).

(**d**) Transcription factors (TF) with binding motifs enriched in regions with age-altered accessibility.

(**e**) Gene expression of indicated TFs. Young (n = 4), Old (n = 8). Data are represented as mean ± SD, Wald test (two-sided).

(**f**) Composite plots of histone modifications (H3K4me3, H3K27me3, and H3K27ac) at Batf and Spi1 related DARs.

(**g**) Composite plot of H3K27ac generated with all H3K27c peaks at non-promoter regions.

(**h**) Distribution of DARs relative to TSS.

(**i**) Composite plots of chromatin accessibility at up (left panel) or down (right panel) regulated TEs with each replicate shown separately.

# Supplementary Fig. 2

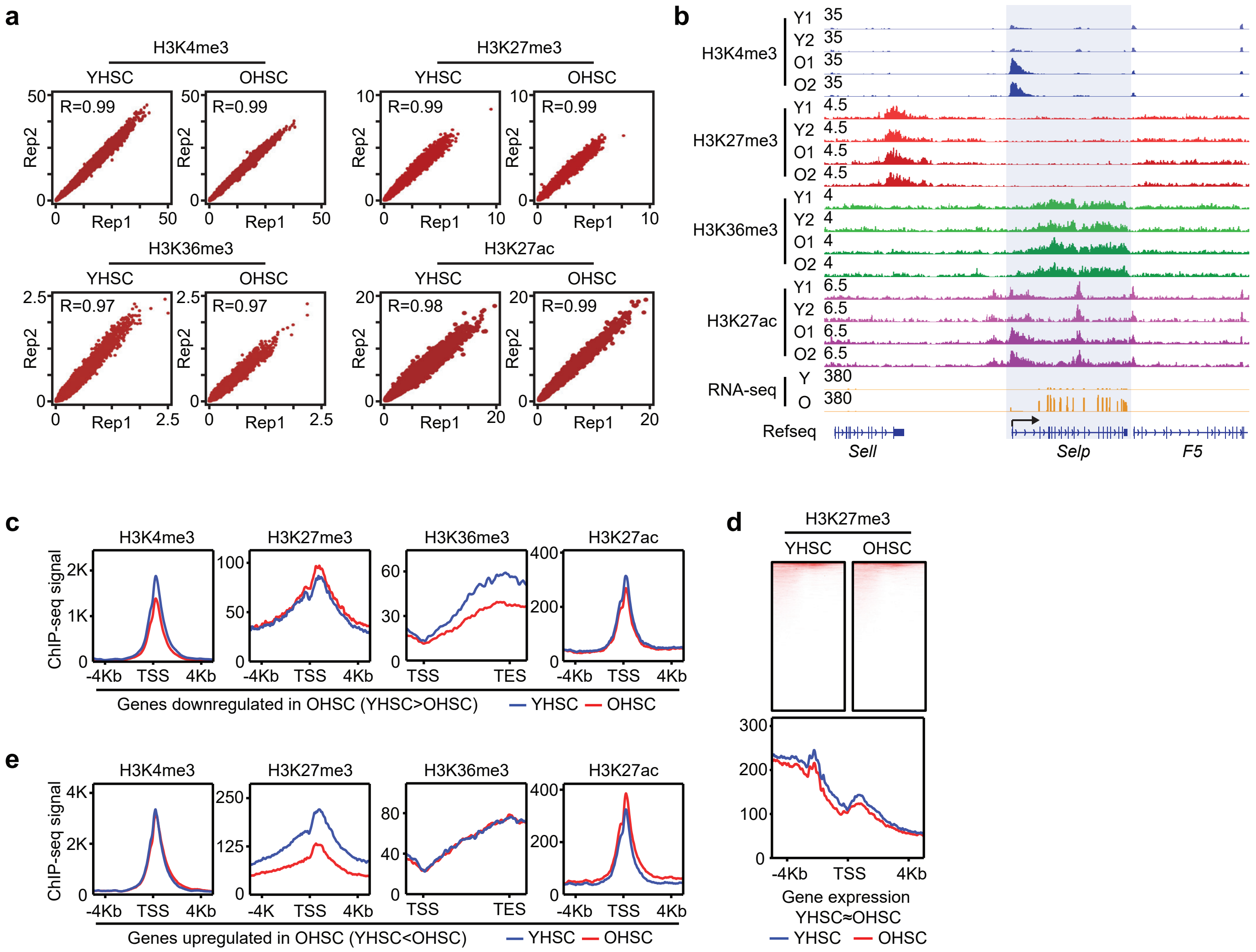

**Supplementary Fig. 2: Quality control of ChIP-seq data from young and old HSC samples.**

(**a**) Scatter plots visualizing the correlation between two biological replicates of indicated histone modification ChIP-seqs generated with young or old HSCs. Pearson correlations are noted.

(**b**) Histone modification and gene expression tracks at *Selp* locus with each replicate of histone modification shown separately.

(**c**) Composite plots of histone modifications generated with genes whose expression was downregulated in OHSCs (FC > 1.5 and FDR < 0.05).

(**d**) Heatmap and composite plots of H3K27me3 generated with genes whose expression level was similar in young and old HSCs (FC < 1.05).

(**e**) Composite plots of histone modifications generated with genes whose expression was upregulated in OHSCs (FC > 1.5 and FDR < 0.05).

# Supplementary Fig. 3

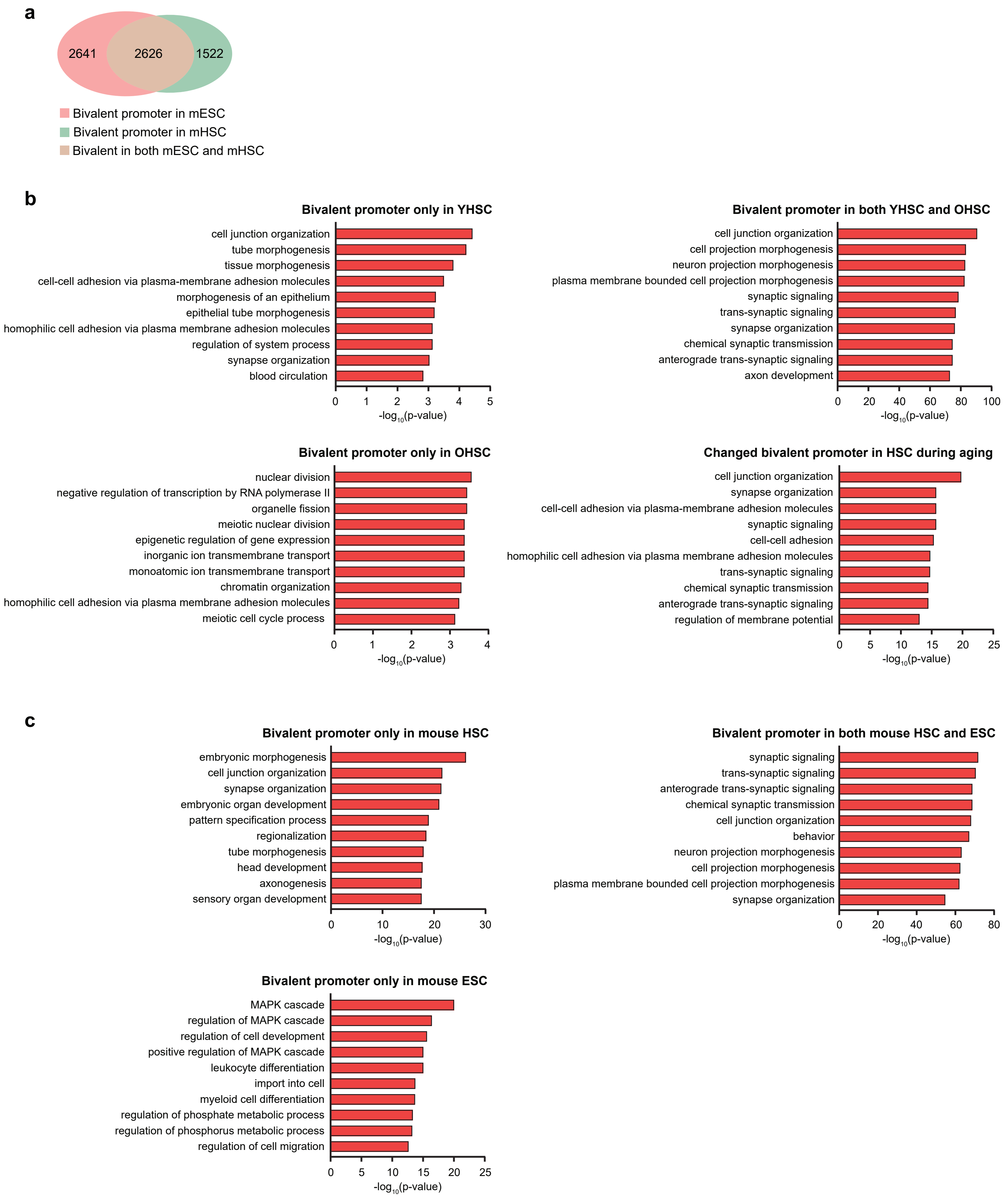

**Supplementary Fig. 3: Pathway analysis of bivalent promoters in young and old HSCs and ESCs.**

(**a**) Venn diagram displaying the number of genes with bivalent promoters in mouse HSCs and ESCs[1, 2].

(**b** and **c**) Pathway analysis of bivalent promoters in young and old HSCs (b), HSCs and ESCs (c). Pathway enrichment analysis was performed using g:Profiler. Enrichment p-values were calculated using a one-sided hypergeometric test and corrected for multiple testing using the g:Profiler g:SCS method.

## Supplementary Fig. 4

**a**

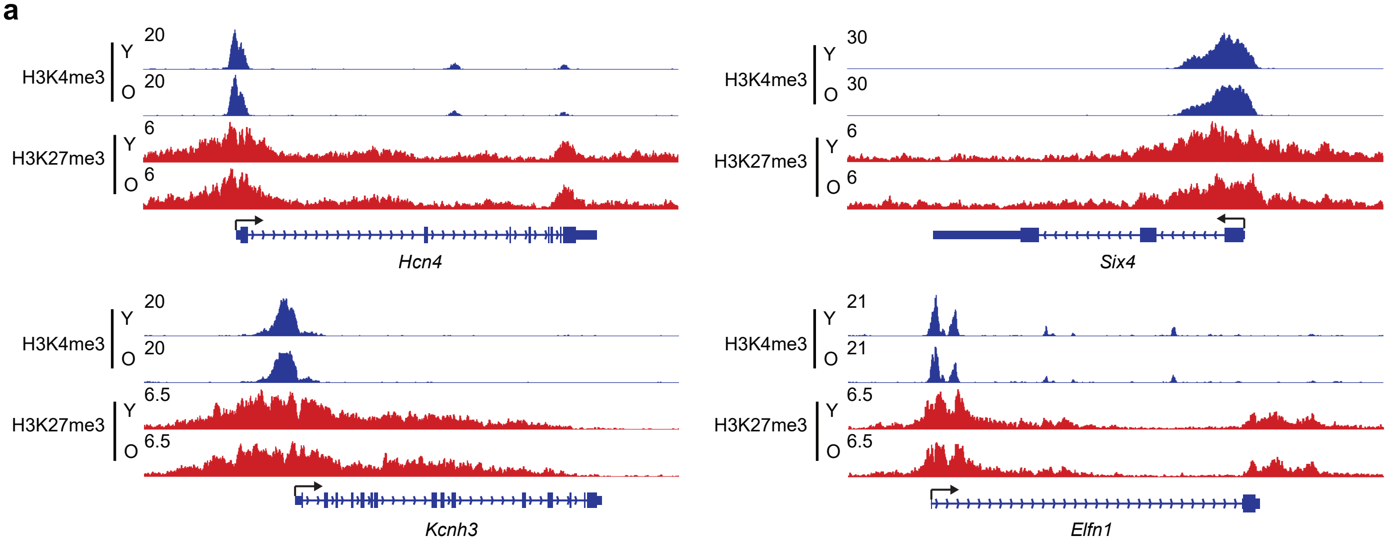


**b**

| | Group 1 | Group 2 | Group 3 | Group 4 | Group 5 | Group 6 | Group 7 | Group 8 |
|---|---|---|---|---|---|---|---|---|
| Change of H3K4me3 in OHSC | Up | Up | Up | Down | Down | Down | No | No |
| Change of H3K27me3 in OHSC | Up | Down | No[1] | Up | Down | No | Up | Down |

[1] Change below cutoff (FC < 1.5)

**Supplementary Fig. 4: Examples and classification of promoter bivalency changes during HSC aging.**

(**a**) Examples of genes with bivalency preserved during HSC aging.

(**b**) Eight groups defined by change of promoter bivalency. The direction of change was shown. FC > 1.5 was used as cutoff for up and down changes. No indicates FC < 1.5.

## Supplementary Fig. 5

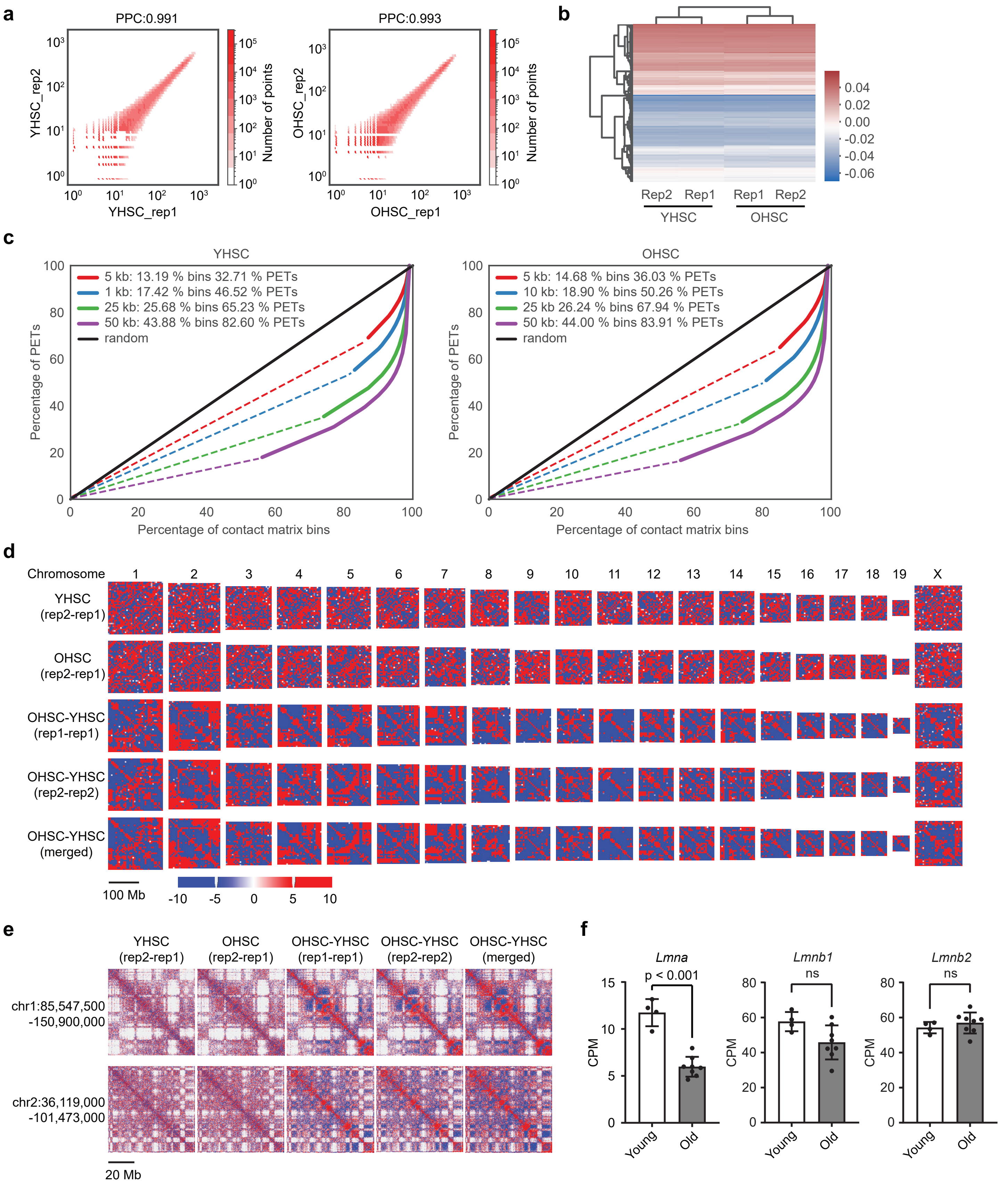

**Supplementary Fig. 5: Quality control of Hi-C data and analysis of chromatin organization changes between young and old HSCs.**

(**a**) Density scatter plots show the correlation between biological replicates of Hi-C data at 100 kb resolution. Pearson correlation coefficient (PPC) was shown on top of each plot.

(**b**) Unsupervised hierarchical clustering of compartments at 100 kb resolution.

(**c**) Resolution estimation plots for Hi-C datasets of young and old HSCs.

(**d**) Comparison of indicated Hi-C contact matrices of each chromosome, plotted at 5 Mb resolution with Juicebox. First two rows: red, rep1 < rep2; blue, rep1 > rep2. Last three rows: red, YHSC < OHSC; blue, YHSC > OHSC.

(**e**) Comparison of Hi-C contact matrices for chromosomal subregions at 250 kb resolution using Juicebox. Left two panels: red, rep1 < rep2; blue, rep1 > rep2. Right three panels: red, YHSC < OHSC; blue, YHSC > OHSC.

(**f**) Expression levels of *Lmna*, *Lmnb1* and *Lmnb2*. Young (n = 4), Old (n = 8). Data are represented as mean ± SD, Wald test (two-sided). ns, not significant.

## Supplementary Fig. 6

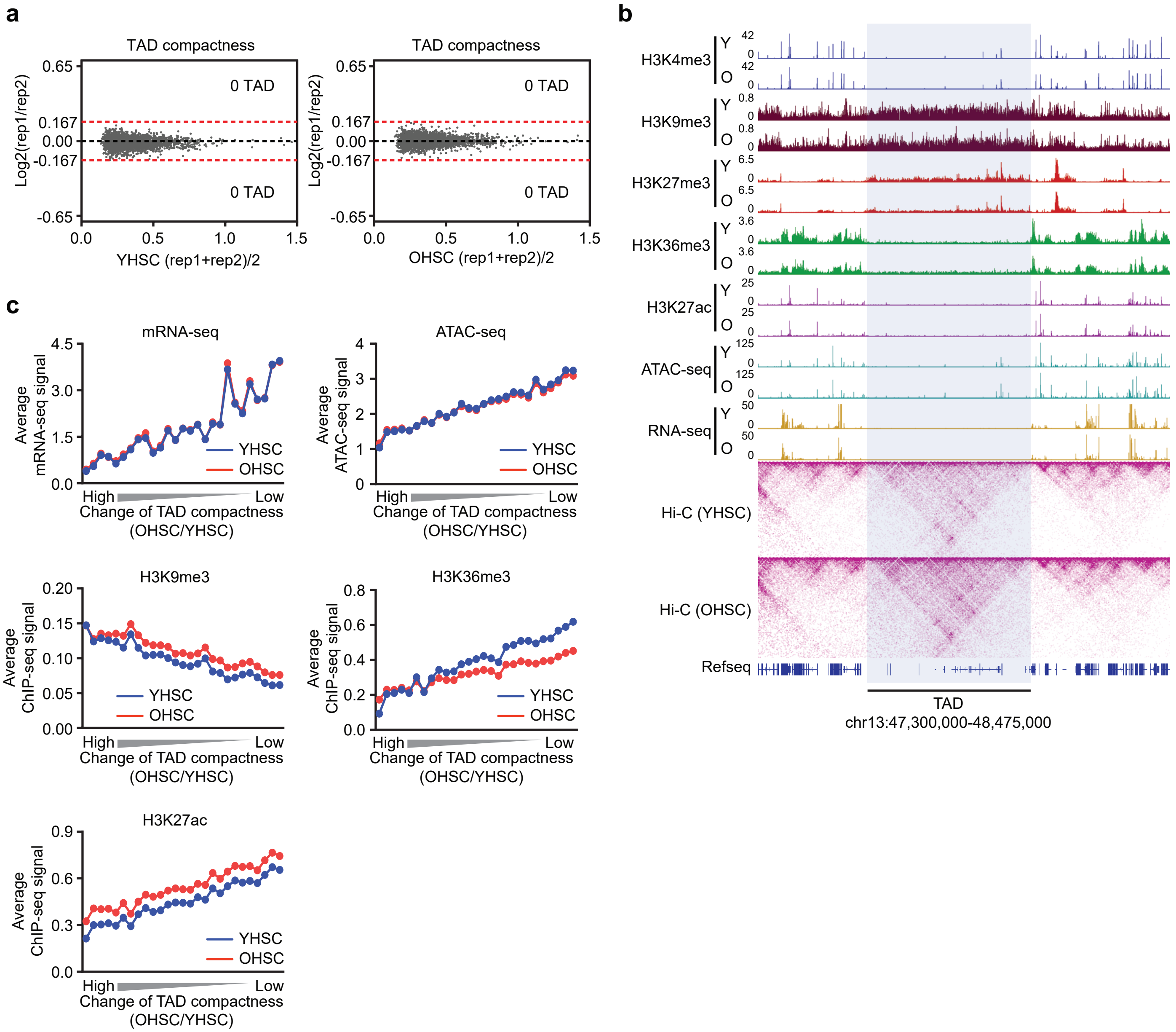

**Supplementary Fig. 6: Analysis of TAD changes in young and old HSCs.**

(**a**) Comparison of TAD compactness between biological replicates of young (left panel) or old (right panel) HSCs to determine the cutoff for changed TADs.

(**b**) WashU Epigenome Browser view of an example of TAD (10 kb resolution) with increased compactness in OHSCs and IGV view of the corresponding chromatin state at this TAD (chr13:47300000-48475000).

(**c**) mRNA-seq, ATAC-seq, H3K9me3, H3K36me3 and H3K27ac signal at TADs (bin size = 100 TADs) sorted by change of compactness (OHSC/YHSC) from high to low.

## Supplementary Fig. 7

**a**

**Top 5 changed TADs**

| TAD location | RPKM (YHSC) | RPKM (OHSC) | Fold change | p-value |
|---|---|---|---|---|
| chr19:36850000-37350000 | 0.195 | 0.281 | 1.44 | 0.037 |
| chr14:37150000-38750000 | 0.195 | 0.242 | 1.24 | 0.002 |
| chr13:47300000-48475000 | 0.193 | 0.235 | 1.22 | 0.018 |
| chr11:55525000-57450000 | 0.178 | 0.211 | 1.19 | 0.001 |
| chr12:26525000-28475000 | 0.191 | 0.223 | 1.16 | 0.036 |

**b**

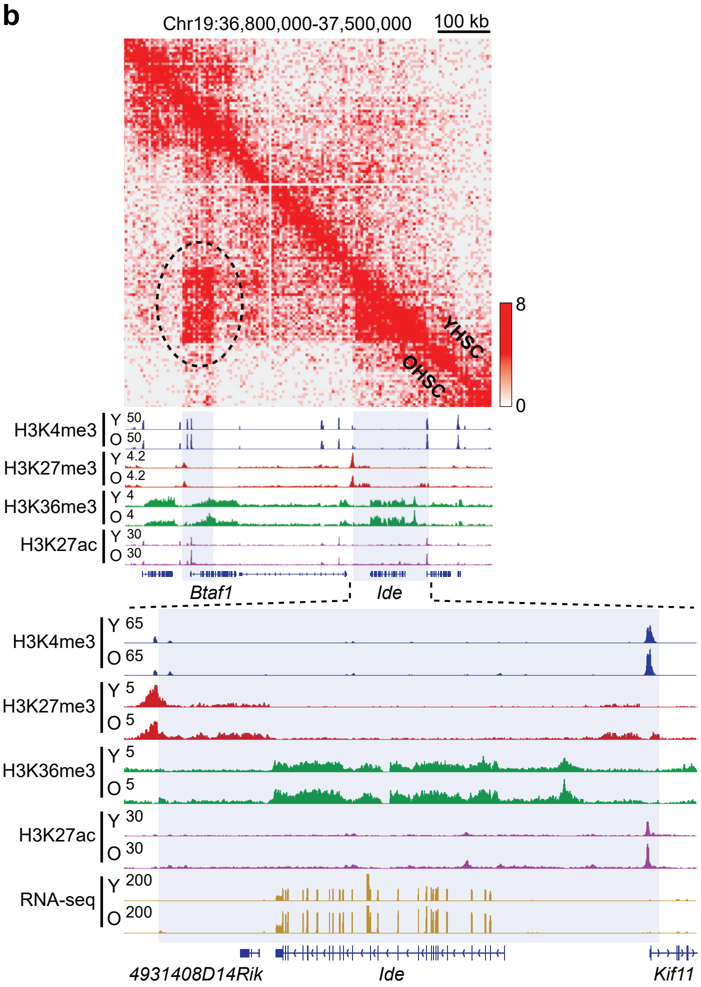


**c** **d**

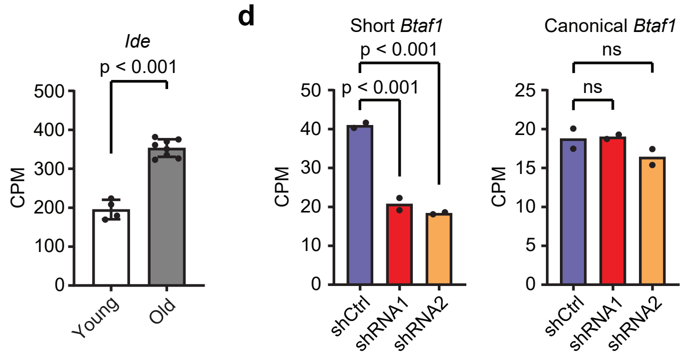


**e**

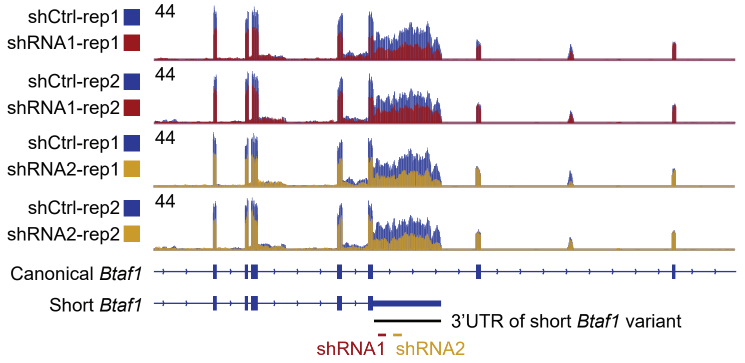


**f**

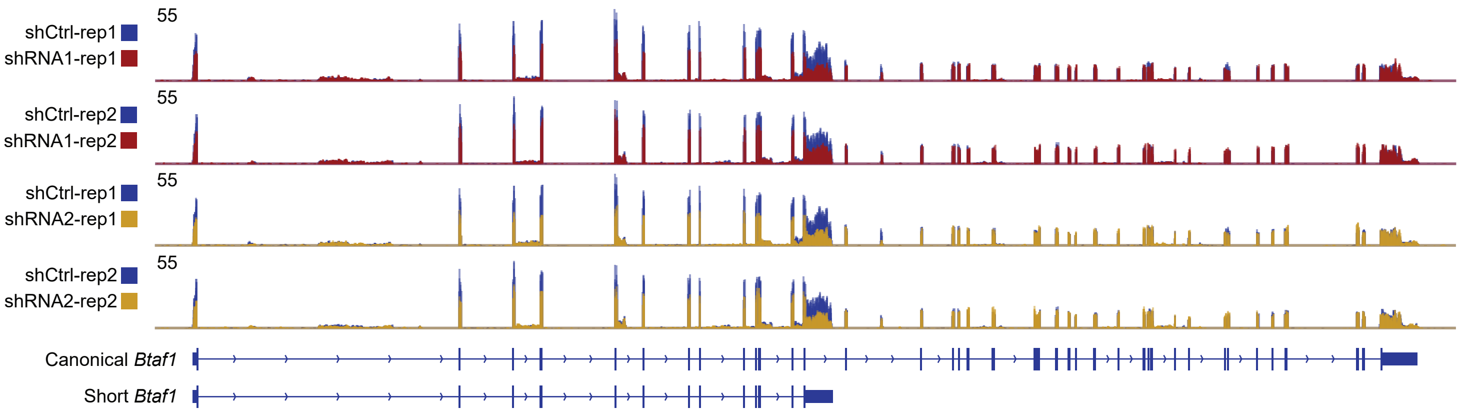


**g**

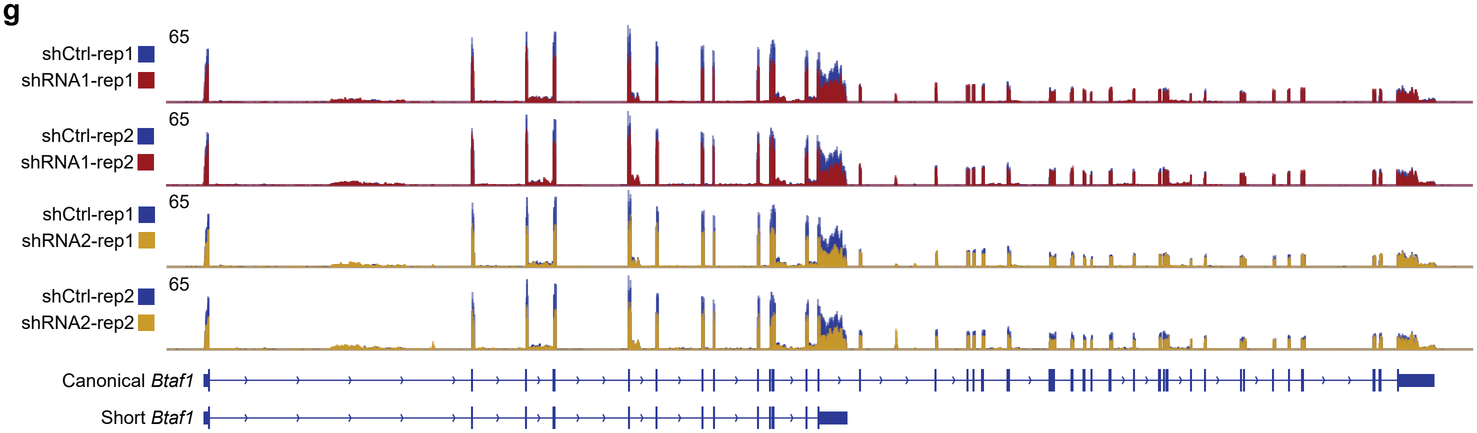

**Supplementary Fig. 7: Analysis of top altered TADs in OHSCs and functional assessment of *nBtaf1* and *Ide* expression.**

(**a**) Top 5 changed TADs during HSC aging. Welch's t-test was applied, n = 2.

(**b**) Hi-C heatmaps showed a new loop formed between part of *Btaf1* and the whole *Ide* in OHSCs. Upper: Hi-C heatmap plotted at 5 kb resolution with Juicebox, the new loop in OHSCs was highlighted with dotted circle; middle: histone modification tracks with the genome locations that form the new loop highlighted. Bottom: expanded view of the histone modification and gene expression tracks at *Ide* locus.

(**c**) Expression levels of *Ide* in young and old HSCs. Young (n = 4), Old (n = 8). Data are represented as mean ± SD, Wald test (two-sided).

(**d**) Quantification of the shorter and canonical *Btaf1* variants in cultured control and knockdown HSCs. HSCs from old mouse were sorted into PVA-based media, then infected with shRNA viruses targeting the shorter *Btaf1* variant. GFP positive cells were sorted for RNA-seq 3.5 days after virus transduction. shCtrl (n = 2), shRNA1 (n = 2), shRNA2 (n = 2). Wald test (two-sided). ns, not significant.

(**e**) Gene expression tracks around 3'UTR of the shorter *Btaf1* variant (*in vitro* knockdown).

(**f**) Gene expression tracks of the shorter and canonical *Btaf1* variants (*in vitro* knockdown).

(**g**) Gene expression tracks of the shorter and canonical *Btaf1* variants (*in vivo* knockdown).

## Supplementary Fig. 8

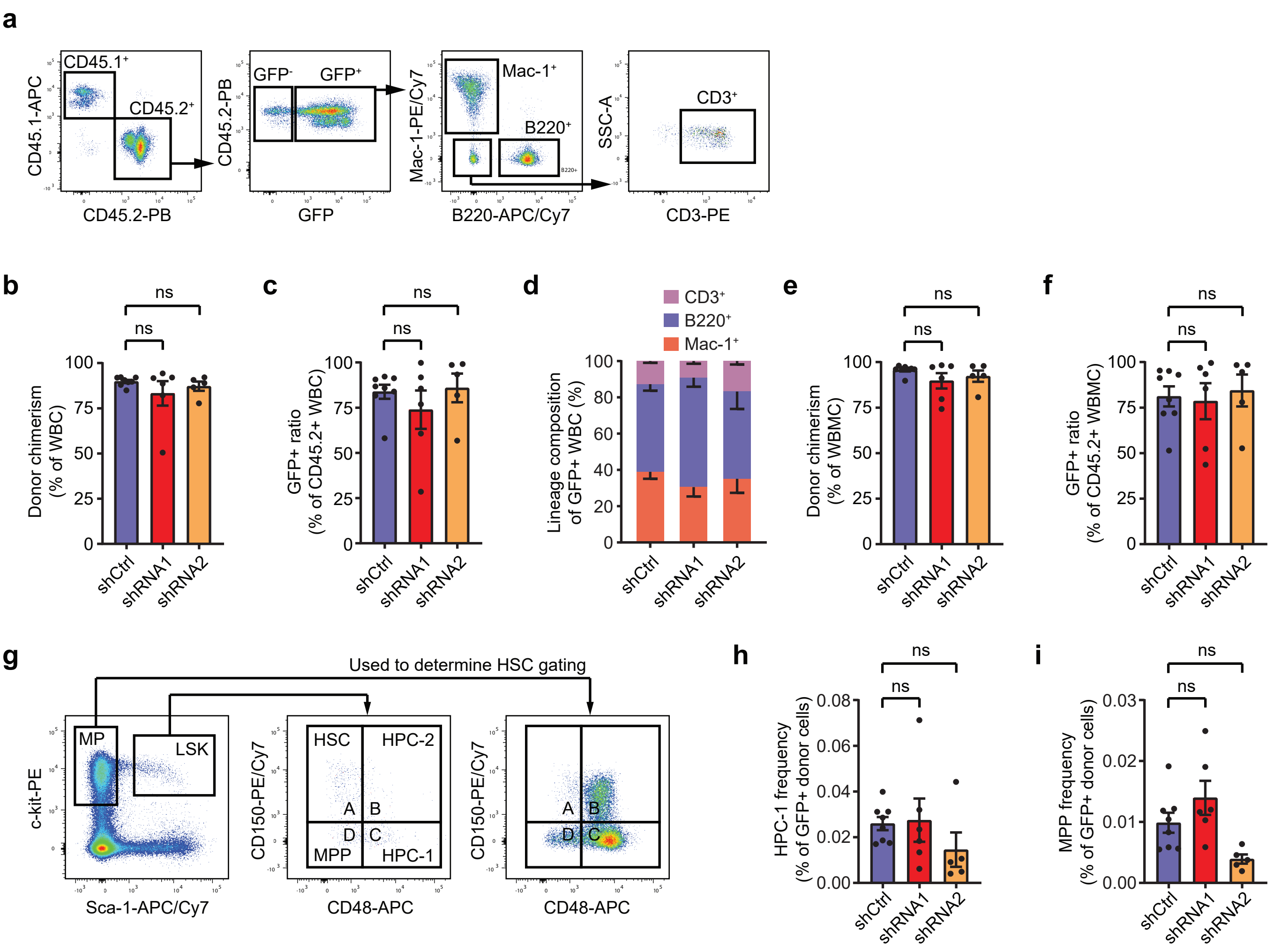

**Supplementary Fig. 8: Peripheral blood (PB) and whole bone marrow (WBM) analysis of recipient mice transplanted with control or *nBtaf1* knockdown HSCs.**

(**a**) Gating strategy for PB analysis of transplanted mice.

(**b**) PB donor chimerism of recipient mice transplanted with control or knockdown HSCs. WBC, white blood cells. shCtrl (n = 8), shRNA1 (n = 6), shRNA2 (n = 5). Data are represented as mean ± SEM, ordinary one-way ANOVA. ns, not significant. Source data are provided as a Source Data file.

(**c**) GFP+ ratio of donor derived white blood cells. shCtrl (n = 8), shRNA1 (n = 6), shRNA2 (n = 5). Data are represented as mean ± SEM, ordinary one-way ANOVA. Source data are provided as a Source Data file.

(**d**) Lineage composition of donor derived GFP+ white blood cells. shCtrl (n = 8), shRNA1 (n = 6), shRNA2 (n = 5). Data are represented as mean ± SEM, two-way ANOVA. Source data are provided as a Source Data file.

(**e**) WBM donor chimerism of recipient mice transplanted with control or knockdown HSCs. WBMC, whole bone marrow cells. shCtrl (n = 8), shRNA1 (n = 6), shRNA2 (n = 5). Data are represented as mean ± SEM, ordinary one-way ANOVA. Source data are provided as a Source Data file.

(**f**) GFP+ ratio of donor derived whole bone marrow cells. shCtrl (n = 8), shRNA1 (n = 6), shRNA2 (n = 5). Data are represented as mean ± SEM, ordinary one-way ANOVA. Source data are provided as a Source Data file.

(**g**) Gating strategy for WBM analysis of transplanted mice.

(**h** and **i**) HPC-1 (h) and MPP (i) frequency in control and knockdown donor cells. shCtrl (n = 8), shRNA1 (n = 6), shRNA2 (n = 5). Data are represented as mean ± SEM, ordinary one-way ANOVA. Source data are provided as a Source Data file.

## Supplementary Fig. 9

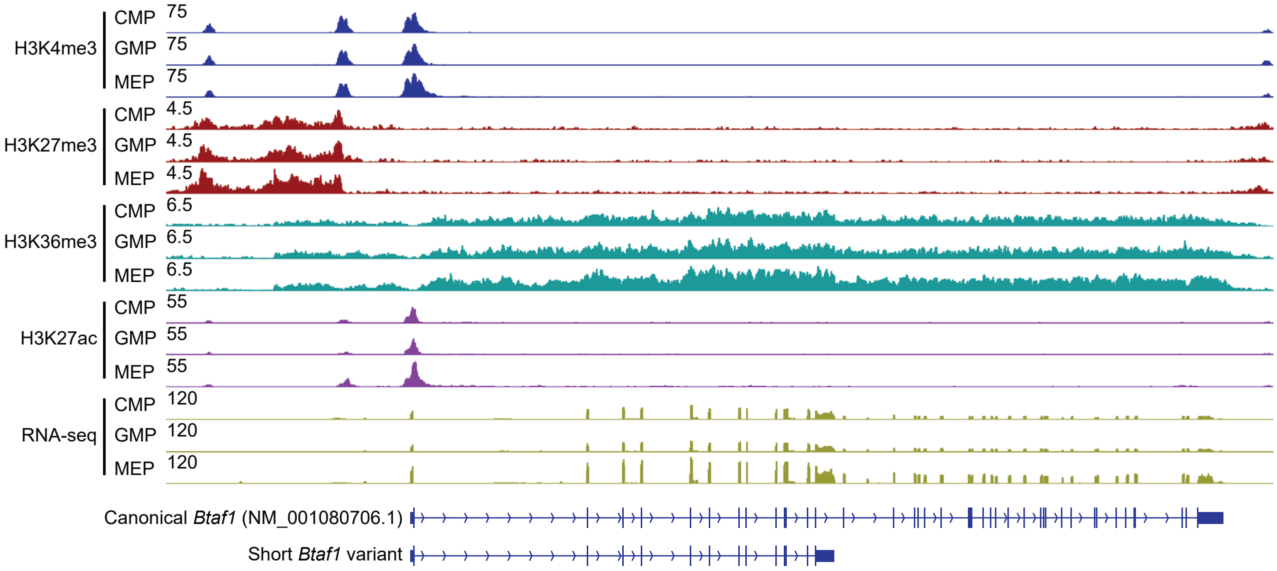

**Supplementary Fig. 9: Histone modifications and expression of *nBatf1* in CMPs, GMPs, and MEPs.**

## Supplementary Fig. 10

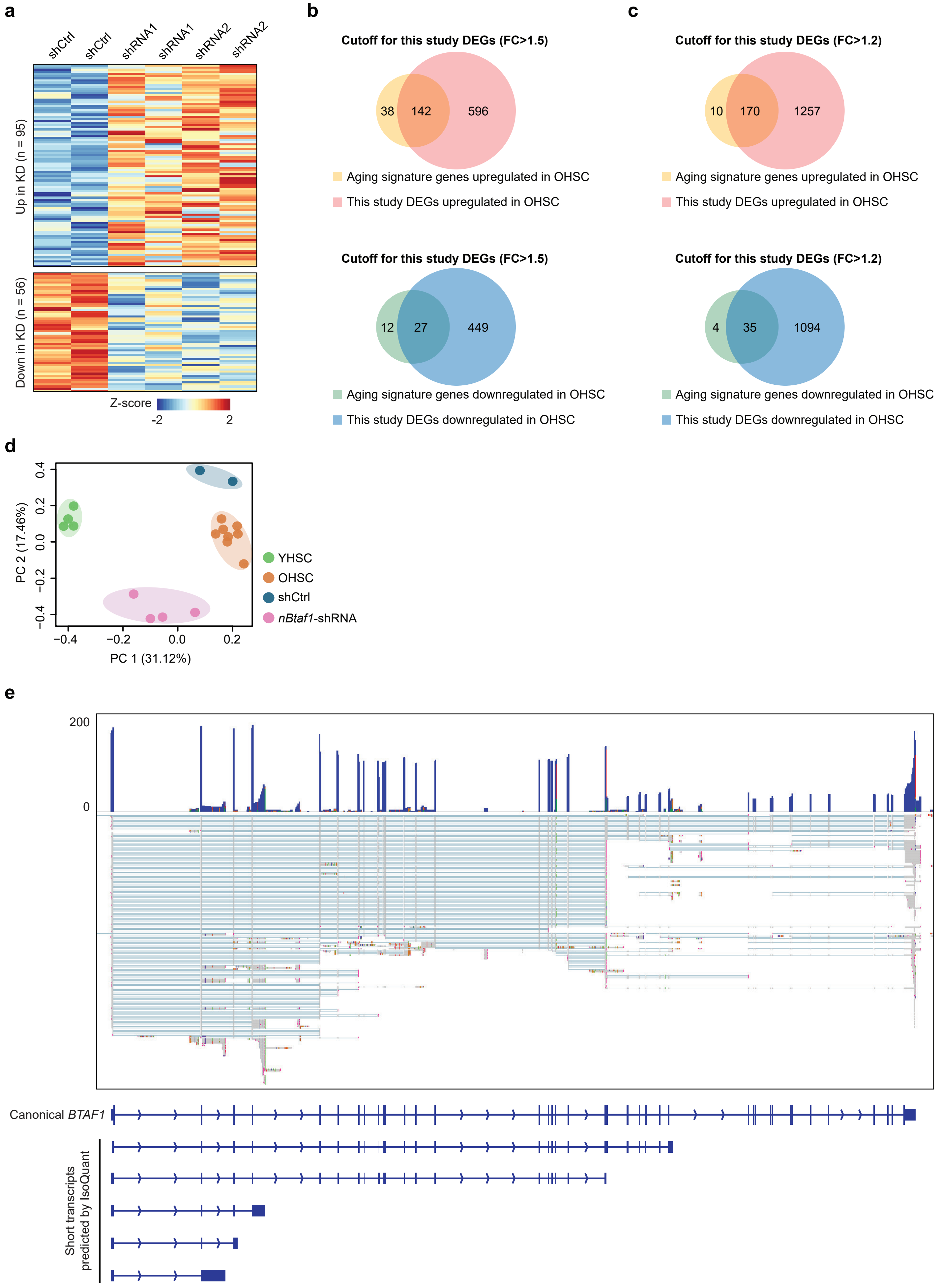

**Supplementary Fig. 10: DEGs after *nBtaf1* knockdown and comparison of aging signature genes and DEGs defined in this study with different cutoff.**

(**a**) Heatmap of DEGs after *nBtaf1* knockdown (FC >1.5, p-value < 0.05). RNA-seq data of HSCs purified from transplanted mice were used. Control HSCs, n = 2; knockdown HSCs, n = 4.

(**b** and **c**) Venn plots of aging signature genes and DEGs (young vs old HSC comparison) defined in this study with cutoff FC >1.5 (b) or FC > 1.2 (c), FDR < 0.05.

(**d**) PCA of mRNA-seq data from young (n = 4), old (n = 8), and in vivo control (n = 2) and *nBtaf1* knockdown (n = 4) HSCs.

(**e**) IGV view of *BTAF1* variants in human immune cells. Nanopore sequencing data of human immune cells was used. Short *BTAF1* transcripts were predicted by IsoQuant[3].

## References


1. Stevens, T.J. *et al*. 3D structures of individual mammalian genomes studied by single-cell Hi-C. *Nature* **544**, 59-64 (2017).
2. Cruz-Molina, S. *et al*. PRC2 Facilitates the Regulatory Topology Required for Poised Enhancer Function during Pluripotent Stem Cell Differentiation. *Cell Stem Cell* **20**, 689-705.e689 (2017).
3. Prjibelski, A.D. *et al*. Accurate isoform discovery with IsoQuant using long reads. *Nat. Biotechnol.* **41**, 915-918 (2023).
