# Supplementary material for: Chromatin reorganization drives overexpression of a Btaf1 variant underpinning hematopoietic aging
Source: Nat Commun. 2026 Mar 18;17:4129. doi: 10.1038/s41467-026-70787-4 (PMC13149550; doi:10.1038/s41467-026-70787-4)
Supplement: Supplementary file 2 — Description of Additional Supplementary Files [file 41467_2026_70787_MOESM2_ESM.pdf]

Description of Additional Supplementary Files

Supplementary Data 1: Differentially accessible regions between young and old HSCs
Differential accessibility was analyzed using DESeq2. Statistical significance was assessed using two-sided Wald tests, and p-values were adjusted for multiple testing using the Benjamini–Hochberg false discovery rate (FDR) method. Significantly changed genes were defined as those with an absolute fold change > 1.5 and FDR < 0.01. ATAC-seq data of HSCs purified from young (n = 4) and old (n = 8) mice were used.

Supplementary Data 2: Differentially expressed genes between young and old HSCs
Differential expression was analyzed using DESeq2. Statistical significance was assessed using two-sided Wald tests, and p-values were adjusted for multiple testing using the Benjamini–Hochberg false discovery rate (FDR) method. Significantly changed genes were defined as those with an absolute fold change > 1.5 and FDR < 0.05. RNA-seq data of HSCs purified from young (n = 4) and old (n = 8) mice were used.

Supplementary Data 3: List of genes with bivalent promoter in HSCs

Supplementary Data 4: QC of Hi-C data

Supplementary Data 5: Information of genes in the top 5 changed TADs
Differential expression was analyzed using DESeq2. Statistical significance was assessed using two-sided Wald tests, and p-values were adjusted for multiple testing using the Benjamini–Hochberg false discovery rate (FDR) method. RNA-seq data of HSCs purified from young (n = 4) and old (n = 8) mice were used.
