# Supplementary material for: Chromatin reorganization drives overexpression of a Btaf1 variant underpinning hematopoietic aging
Source: Nat Commun. 2026 Mar 18;17:4129. doi: 10.1038/s41467-026-70787-4 (PMC13149550; doi:10.1038/s41467-026-70787-4)
Supplement: Supplementary file 8 — Reporting Summary [file 41467_2026_70787_MOESM8_ESM.pdf]

nature portfolio

Corresponding author(s): *Double-anonymous peer review submissions: write DAPR and your manuscript number here instead of author names.*

Last updated by author(s): *YYYY-MM-DD*

Reporting Summary

Nature Portfolio wishes to improve the reproducibility of the work that we publish. This form provides structure for consistency and transparency in reporting. For further information on Nature Portfolio policies, see our Editorial Policies and the Editorial Policy Checklist.

Statistics

For all statistical analyses, confirm that the following items are present in the figure legend, table legend, main text, or Methods section.

n/a	Confirmed	
☐	☒	The exact sample size (*n*) for each experimental group/condition, given as a discrete number and unit of measurement
☐	☒	A statement on whether measurements were taken from distinct samples or whether the same sample was measured repeatedly
☐	☒	The statistical test(s) used AND whether they are one- or two-sided<br>*Only common tests should be described solely by name; describe more complex techniques in the Methods section.*
☐	☒	A description of all covariates tested
☐	☒	A description of any assumptions or corrections, such as tests of normality and adjustment for multiple comparisons
☒	☐	A full description of the statistical parameters including central tendency (e.g. means) or other basic estimates (e.g. regression coefficient) AND variation (e.g. standard deviation) or associated estimates of uncertainty (e.g. confidence intervals)
☐	☒	For null hypothesis testing, the test statistic (e.g. *F*, *t*, *r*) with confidence intervals, effect sizes, degrees of freedom and *P* value noted<br>*Give P values as exact values whenever suitable.*
☒	☐	For Bayesian analysis, information on the choice of priors and Markov chain Monte Carlo settings
☒	☐	For hierarchical and complex designs, identification of the appropriate level for tests and full reporting of outcomes
☒	☐	Estimates of effect sizes (e.g. Cohen's *d*, Pearson's *r*), indicating how they were calculated

Our web collection on statistics for biologists contains articles on many of the points above.

Software and code

Policy information about availability of computer code

Data collection

FACS data was collected on BD FACS Aria II, Fusion using Diva software. Hi-C, ATAC-seq, ChIP-seq and RNA-seq libraries were sequenced on Illumina HiSeq 2500, NextSeq 2000 or NovaSeq 6000 System.

Data analysis

FACS Analysis was performed in FlowJo v10 and statistics data was generated in GraphPad Prism 9.

Hi-C analysis: Raw Hi-C reads in FASTQ files were aligned to reference genome mm10 by HiCUP (v 0.7.2). High-quality unique paired-end tags (PETs) (MAPQ >= 10 from HiCUP) were further converted to HIC files by Juicer (v1.6.0) for visualization and cLoops2 (v0.0.3) data directories for quantification, similarity, and aggregation analysis. Hi-C compartment eigenvectors were calculated by hicPCA from HiCExplorer (v3.6) at the resolution of 100 kb, and TADs were called by Juicer at the resolution of 25 kb.

ATAC-seq analysis: All sequencing reads were trimmed using cutadapt, and trimmed reads (>36 bp minimum alignment length) were mapped against the mm10 genome using BWA aligner. We used de-duplicated and uniquely mapped reads for peak calling analysis after excluding high-sensitive black-list regions defined by ENCODE. The candidate peaks were predicted by MACS peak calling software (FDR < 0.05). After identifying narrow peaks from young and old HSC replicates, we created a merged set of consensus peaks and generated a matrix of open chromatin regions (OCRs). This OCR matrix was then imported into the R package DESeq2, and we determined differentially accessible regions (DARs) with cutoff: FC > 1.5, CPM > 1.5, FDR < 0.01. Finally, the candidate differential open chromatin regions were submitted to search for potential transcription factor binding sites using HOMER software with non-DARs as background regions. In this analysis, de novo motif and known motif searches were performed, and we reported the top five significant de novo motif results. Composite plots and heatmaps were generated by customized Python script and Java TreeView software.

ChIP-seq analysis: All sequencing reads were trimmed using cutadapt, and trimmed reads (>36 bp minimum alignment length) were mapped

against the mm10 genome using BWA aligner. We used de-duplicated and uniquely mapped reads for peak calling analysis after excluding high-sensitive black-list regions defined by ENCODE. For H3K4me3 and H3K27ac histone marks, the narrow candidate peaks were predicted by MACS peak calling software (FDR < 0.05). The sequencing libraries of all the histone marks were normalized (10 M reads), and we summarized average peak size to 1 kb binning across all mouse genomes. These normalized files were used to perform broad peak calling and chromatin state analysis (e.g. IDEAS). For H3K27me3, we identified broad peaks from IDEAS chromatin states (FDR < 0.05) and expanded the peak range to include signals that appeared significant compared to random background height (average broad peak height > 0.3, and significant p-val < 1e-4). To identify histone peaks that changed between age groups, we used cutoff FC > 1.5 (merged data) and each replicate from one age group should have an FC of at least 1.2 compared to either replicate from the other age group. To analyze bivalent promoters, we first checked whether an H3K4me3 or H3K27me3 peak could be assigned to a gene promoter. For H3K4me3 peaks, if the peak was within 1 kb of the transcription start site (TSS) of a gene, it was assigned to this gene. For H3K27me3 peaks, if the peak overlapped with TSS or within 2 kb downstream of the TSS of a gene, it was assigned to this gene. A gene is defined in this study as having a bivalent promoter if there are both H3K4me3 and H3K27me3 peaks assigned to this gene. Composite plots and heatmaps were generated with customized Python script and Java TreeView software.

RNA-seq analysis: For analysis of transcriptome datasets, we built an index for STAR using the GENCODE M22 reference feature including protein-coding and non-coding genes. Prior to sequence alignment, we applied trim galore (version 0.4.3) with cutadapt (version 1.12) to remove any unnecessary genomic fragments (e.g. adapter dimers) and low-quality nucleotide sequences from the raw reads. We mapped adapter trimmed sequencing reads to the mouse reference genome (mm10) using STAR aligner and calculated the raw count using featureCounts software (gene-level). Differentially expressed gene (DEG) lists were generated with DESeq2 using cutoff: FC > 1.5, FDR < 0.05. For transposable element (TE) detection, we utilized SQuIRE and used limma based edgeR package of R to find differential transposable elements.

Code availability: All customized Python scripts used in the manuscript are available via GitHub repository URL (https://github.com/genomicspark/ESCA_Unit_Scripts) and has been archived in Zenodo for citation (https://doi.org/10.5281/zenodo.17857712).

For manuscripts utilizing custom algorithms or software that are central to the research but not yet described in published literature, software must be made available to editors and reviewers. We strongly encourage code deposition in a community repository (e.g. GitHub). See the Nature Portfolio guidelines for submitting code & software for further information.

Data

Policy information about availability of data

All manuscripts must include a data availability statement. This statement should provide the following information, where applicable:

- Accession codes, unique identifiers, or web links for publicly available datasets
- A description of any restrictions on data availability
- For clinical datasets or third party data, please ensure that the statement adheres to our policy

The raw data generated in this study have been deposited in the NCBI Gene Expression Omnibus database under accession code GSE204933 (https://www.ncbi.nlm.nih.gov/geo/query/acc.cgi?acc= GSE204933).

Research involving human participants, their data, or biological material

Policy information about studies with human participants or human data. See also policy information about sex, gender (identity/presentation), and sexual orientation and race, ethnicity and racism.

Reporting on sex and gender	N/A
Reporting on race, ethnicity, or other socially relevant groupings	N/A
Population characteristics	N/A
Recruitment	N/A
Ethics oversight	N/A

Note that full information on the approval of the study protocol must also be provided in the manuscript.

Field-specific reporting

Please select the one below that is the best fit for your research. If you are not sure, read the appropriate sections before making your selection.

☒ Life sciences ☐ Behavioural & social sciences ☐ Ecological, evolutionary & environmental sciences

For a reference copy of the document with all sections, see nature.com/documents/nr-reporting-summary-flat.pdf

Life sciences study design

All studies must disclose on these points even when the disclosure is negative.

Sample size	We used power calculations from previous experiments to determine the sample size.
Data exclusions	No data were excluded from the analyses.
Replication	Biological replicates of each experiments were described in the corresponding figure legends.
Randomization	The mice used in this study were randomly allocated to different experiment group.
Blinding	The investigators were blinded to group allocation during data analysis.

Reporting for specific materials, systems and methods

We require information from authors about some types of materials, experimental systems and methods used in many studies. Here, indicate whether each material, system or method listed is relevant to your study. If you are not sure if a list item applies to your research, read the appropriate section before selecting a response.

Materials & experimental systems

n/a	Involved in the study	
☐	☒	Antibodies
☒	☐	Eukaryotic cell lines
☒	☐	Palaeontology and archaeology
☐	☒	Animals and other organisms
☒	☐	Clinical data
☒	☐	Dual use research of concern
☒	☐	Plants

Methods

n/a	Involved in the study	
☐	☒	ChIP-seq
☐	☒	Flow cytometry
☒	☐	MRI-based neuroimaging

Antibodies

Antibodies used	Flow cytometry. The following antibodies are from Biolegend: Biotin anti-TER119 (Cat# 116204, 1:100 dilution), Biotin anti-B220 (Cat# 103204, 1:100 dilution), Biotin anti-CD3 (Cat# 100244, 1:100 dilution), Biotin anti- Mac1 (Cat# 101204, 1:100 dilution), Biotin anti-Gr1 (Cat# 108404, 1:100 dilution), Biotin anti-IL7Rα (Cat# 135005, 1:100 dilution), PB anti-TER119 (Cat# 116232, 1:200 dilution), PB anti-B220 (Cat# 103227, 1:200 dilution), PB anti- Mac1 (Cat# 101224, 1:200 dilution), PB anti-CD3 (Cat# 100214, 1:200 dilution), BV421 anti-Gr1 (Cat# 108445, 1:200 dilution), BV421 anti-IL7Rα (Cat# 135027, 1:200 dilution), APC/Cy7 anti-Sca1 (Cat# 108126, 1:200 dilution), PE anti- cKit (Cat# 105808, 1:200 dilution), APC anti-Flk2 (Cat# 135310, 1:50 dilution), PE/Cy7 anti-CD150 (Cat# 115914, 1:200 dilution), APC anti-CD48 (Cat# 103412, 1:200 dilution), APC anti-CD45.1 (Cat# 110714, 1:200 dilution), PB anti-CD45.2 (Cat# 109820, 1:100 dilution), PerCP/Cy5.5 anti-TER119 (Cat# 116228, 1:200 dilution), APC/Cy7 anti-CD45R/B220 (Cat# 103224, 1:200 dilution), PE anti-CD3 (Cat# 100206, 1:200 dilution), PE/Cy7 anti- Mac1 (Cat# 101216, 1:200 dilution), BV510 anti-Gr1 (Cat# 108457, 1:200 dilution), APC anti-CD41 (Cat# 133914, 1:200 dilution), PerCP/Cy5.5 anti-CD45.1 (Cat# 110728, 1:100 dilution). FITC anti-CD34 is from ThermoFischer (Cat# 11-0341-85, 1:50 dilution).<br><br>ChIP–seq. Anti-H3K4me3 (Sigma, Cat# 07-473), anti-H3K9me3 (abcam, Cat# ab8898), anti-H3K27me3 (Diagenode, Cat# C15410195), anti-H3K36me3 (abcam, Cat# ab9050), anti-H3K27ac (abcam, Cat# ab4729).
Validation	All antibodies used in this study are commercially available, and their validation is supported by references provided on the manufacturer's website.

Animals and other research organisms

Policy information about studies involving animals; ARRIVE guidelines recommended for reporting animal research, and Sex and Gender in Research

Laboratory animals	Young C57BL/6J (CD45.2, JAX #:000664) male mice (3-4 months) were purchased from The Jackson Laboratory, old C57BL/6J male mice (24-26 months) were acquired from the NIA Aged Rodent Colony. Young female transplant recipient B6.SJL-Ptprca Pepcb/BoyJ (CD45.1, JAX #:002014) mice were obtained from The Jackson Laboratory. Animals were co-housed in a barrier SPF facility and euthanized by CO2 administration followed by cervical dislocation.
Wild animals	No wild animals were involved in this study.
Reporting on sex	Male C57BL/6J mice were used in this study. For recipient B6.SJL-Ptprca Pepcb/BoyJ mice, female mice were used.

Field-collected samples | This study did not involve samples collected from the field.

Ethics oversight | Institutional Animal Care and Use Committees (National Institute on Aging)

Note that full information on the approval of the study protocol must also be provided in the manuscript.

Plants

Seed stocks | *Report on the source of all seed stocks or other plant material used. If applicable, state the seed stock centre and catalogue number. If plant specimens were collected from the field, describe the collection location, date and sampling procedures.*

Novel plant genotypes | *Describe the methods by which all novel plant genotypes were produced. This includes those generated by transgenic approaches, gene editing, chemical/radiation-based mutagenesis and hybridization. For transgenic lines, describe the transformation method, the number of independent lines analyzed and the generation upon which experiments were performed. For gene-edited lines, describe the editor used, the endogenous sequence targeted for editing, the targeting guide RNA sequence (if applicable) and how the editor was applied.*

Authentication | *Describe any authentication procedures for each seed stock used or novel genotype generated. Describe any experiments used to assess the effect of a mutation and, where applicable, how potential secondary effects (e.g. second site T-DNA insertions, mosiacism, off-target gene editing) were examined.*

ChIP-seq

Data deposition

☒ Confirm that both raw and final processed data have been deposited in a public database such as GEO.

☒ Confirm that you have deposited or provided access to graph files (e.g. BED files) for the called peaks.

Data access links
May remain private before publication. | ChIP-seq data generated by this study have been deposited to GEO with public accession of GSE204933 (https://www.ncbi.nlm.nih.gov/geo/query/acc.cgi?acc= GSE204933).

Files in database submission | *Provide a list of all files available in the database submission.*

Genome browser session
(e.g. UCSC) | https://genomicspark.shinyapps.io/Zong_Park_Epigenetic_Aging

Methodology

Replicates | ChIP-seq in this study were performed with two replicates.

Sequencing depth | Pair-end sequenced ChIP-seq samples (2x51bp for H3K9me3, 2x75bp for other marks):
Samples total_reads mapped uniq_mapped dedup_uniq_mapped
YHSC_H3K4me3_Rep1 91418089 84393318 77826897 37532049
YHSC_H3K4me3_Rep2 92645101 84037941 78704042 26723527
OHSC_H3K4me3_Rep1 101093753 89052961 78739963 36957478
OHSC_H3K4me3_Rep2 105281872 96365530 85737250 38287372
YHSC_H3K27me3_Rep1 101044948 95818014 88576953 64098581
YHSC_H3K27me3_Rep2 99019116 88361980 82108076 50522764
OHSC_H3K27me3_Rep1 95677805 91248261 82343754 56148091
OHSC_H3K27me3_Rep2 111487542 107116939 96984942 60116481
YHSC_H3K36me3_Rep1 101634289 99327390 89789561 77130268
YHSC_H3K36me3_Rep2 97143227 92868082 84927439 65706627
OHSC_H3K36me3_Rep1 92941781 90272041 80462335 70333752
OHSC_H3K36me3_Rep2 108664391 104815101 93585726 79192033
YHSC_H3K27ac_Rep1 90840284 84377787 82077988 38599058
YHSC_H3K27ac_Rep2 95305236 87940904 84913207 24653198
OHSC_H3K27ac_Rep1 106921525 98445122 95716842 45578817
OHSC_H3K27ac_Rep2 92247759 87290733 84853393 40079697
YHSC_H3K9me3_rep1 119546100 116609306 86185591 67421853
YHSC_H3K9me3_rep2 116425758 113566366 83840419 65538803
OHSC_H3K9me3_rep1 121944076 118864082 87059317 68188696
OHSC_H3K9me3_rep2 101026038 98472210 72342775 58122643
YCMP_H3K4me3_rep1 55555826 50791166 46543857 31620703
YCMP_H3K4me3_rep2 61592218 57154116 52183621 31293207
YCMP_H3K27me3_rep1 49549272 46532167 43566640 36761856
YCMP_H3K27me3_rep2 61366462 58281217 54950441 42464401
YCMP_H3K36me3_rep1 61645572 60095303 54053865 48193310
YCMP_H3K36me3_rep2 61661130 60256553 53996308 47297412
YCMP_H3K27ac_rep1 53184768 50871749 48501378 36521246
YCMP_H3K27ac_rep2 61556562 59210156 56540740 39119032
YGMP_H3K4me3_rep1 47459262 43493065 40435313 29664904
YGMP_H3K4me3_rep2 55411860 52165684 48765474 32142788

	YGMP_H3K27me3_rep1 62953428 59409005 55799821 42684780<br>YGMP_H3K27me3_rep2 58546740 55264761 52220478 41347190<br>YGMP_H3K36me3_rep1 53160648 51709241 46999100 42451580<br>YGMP_H3K36me3_rep2 57815810 56497812 51306466 45690213<br>YGMP_H3K27ac_rep1 55022092 53159539 50720242 38271761<br>YGMP_H3K27ac_rep2 49980110 48317826 46189699 32726520<br>YMEP_H3K4me3_rep1 115015622 104917234 96022372 41928637<br>YMEP_H3K4me3_rep2 84922524 77795409 71485757 33304790<br>YMEP_H3K27me3_rep1 91723248 82745555 76995472 44705106<br>YMEP_H3K27me3_rep2 90792420 82526620 77284743 39921597<br>YMEP_H3K36me3_rep1 95425134 91395989 81438374 63143779<br>YMEP_H3K36me3_rep2 205777740 198711924 176990222 107003430<br>YMEP_H3K27ac_rep1 102394960 95450231 91131262 32994737<br>YMEP_H3K27ac_rep2 59626480 55922723 53388095 21300723
Antibodies	Anti-H3K4me3 (Sigma, Cat# 07-473), anti-H3K9me3 (abcam, Cat# ab8898), anti-H3K27me3 (Diagenode, Cat# C15410195), anti-H3K36me3 (abcam, Cat# ab9050), anti-H3K27ac (abcam, Cat# ab4729).
Peak calling parameters	All sequencing reads were trimmed using cutadapt, and trimmed reads (>36 bp minimum alignment length) were mapped against an mm10 genome using BWA aligner. We used de-duplicated and uniquely mapped reads for peak calling analysis after excluding high-sensitive black-list regions defined by ENCODE. For H3K4me3 and H3K27ac histone marks, the narrow candidate peaks were predicted by MACS peak calling software (FDR < 0.05, shift 0, extsize 150). The sequencing libraries of all the histone marks were normalized (10 M reads). These normalized files were used to perform broad peak calling and chromatin state analysis. For H3K27me3 and H3K36me3, we identified broad peaks from IDEAS chromatin states (FDR < 0.05) and expanded the peak range to include any markers that appeared significant compared to random background height (average broad peak height > 0.3, and significant p-val < 1e-4).
Data quality	Strong peaks were confirmed with visualization through genome browser for all samples.
Software	BWA-MEM (v.2.2.1): https://github.com/lh3/bwa<br>IDEAS (v.1.0.0) :https://github.com/seqcode/IDEAS<br>MACS2 (v.2.2.7.1): https://pypi.org/project/MACS2/

Flow Cytometry

Plots

Confirm that:

☒ The axis labels state the marker and fluorochrome used (e.g. CD4-FITC).

☒ The axis scales are clearly visible. Include numbers along axes only for bottom left plot of group (a 'group' is an analysis of identical markers).

☒ All plots are contour plots with outliers or pseudocolor plots.

☒ A numerical value for number of cells or percentage (with statistics) is provided.

Methodology

Sample preparation	For peripheral blood sample preparation, blood samples were treated with ACK twice to remove red blood cells, then stained with antibody cocktail.<br>For whole bone marrow sample preparation, bones from legs and/or arms were crushed and filtered. Then bone marrow cells were treated with ACK once to remove red blood cells then stained with antibody cocktail.<br>For c-kit enrichment and cell sorting, bone marrow cells were positive selected using PE-c-kit antibody and EasySep™ PE Positive Selection Kit II, then stained with antibody cocktail.
Instrument	BD FACS Aria II, BD FACS Fusion.
Software	BD FACSDiva and FlowJo v10 software were used for data acquisition and analysis.
Cell population abundance	Cell purity was assessed after sorting, with more than 98% purity achieved after each sorting.
Gating strategy	Included in Figure 4d, Supplementary Figure 8a and 8g.

☒ Tick this box to confirm that a figure exemplifying the gating strategy is provided in the Supplementary Information.
